# The Challenge of Cardiogenic Shock Transfers: The Devil in the Details

**DOI:** 10.1016/j.jscai.2026.105325

**Published:** 2026-04-07

**Authors:** Michele L. Esposito, Sandeep Nathan

**Affiliations:** a Division of Cardiology, Medical University of South Carolina, Charleston, South Carolina; b Heart & Vascular Center, University of Chicago Medicine, Chicago, Illinois

**Keywords:** acute myocardial infarction, cardiogenic shock, heart failure, intra-aortic balloon pump, temporary mechanical support

For decades, the intra-aortic balloon pump (IABP) has remained the cornerstone of temporary mechanical circulatory support (tMCS) for cardiogenic shock (CS)^1^^,^^2^; however, management of these patients varies greatly between (and even within) community centers (“spokes”) which refer to tertiary care centers (“hubs”) within a classic “hub-and-spoke” model of care. This observation becomes especially relevant, given that approximately 90% of patients with acute myocardial infarction–related CS (AMI-CS) in the US present to community hospitals.^3^ To that end, prior studies have shown mortality differences among CS patients between transferring and tertiary centers, suggesting opportunities for improvements in best practices across systems of care.^4^^,^^5^ However, it is unclear whether this discrepancy persists within a cohort uniformly supported by IABP therapy, which has historically been the most readily available treatment for CS at community centers.

In this issue of *JSCAI*, Sundermeyer et al^6^ present a retrospective analysis from the Cardiogenic Shock Working Group (CSWG) describing clinical characteristics and outcomes of patients with CS supported by IABP at CSWG sites, divided by the location of IABP implant. Between 2020 and 2024, CS patients receiving an IABP as the initial choice of tMCS were identified in the CSWG registry, and further subdivided into patients who were primarily implanted at a tertiary center (CSWG site) vs implanted at a referring (community) center and then ultimately transferred to a tertiary center.

In total, 2112 patients were selected for analysis, including 652 patients (30.9%) transferred from referral centers and 1460 patients (69.1%) from CSWG centers. Shock etiology varied between groups, with transferred patients more likely to be afflicted by AMI-CS compared with HF-CS (57.8% vs 27.1%). Transferred patients had lower rates of comorbidities, but were more likely to present with higher baseline, 24-hour, and maximum SCAI shock stages compared with nontransferred patients, suggesting these patients were further along the hemo-metabolic shock spiral at the time of transfer. In-hospital mortality rates were higher in transferred patients (33.1% vs 26.4%). Additionally, they were more likely to experience major complications, including bleeding requiring transfusion, in-hospital cardiac arrest, limb ischemia, and stroke. Overall, transferred patients were sicker and more likely to present with AMI-CS, suggesting a higher acuity to their initial clinical presentation.

The authors should be commended for conducting a robust analysis of a large, real-world data set to guide further efforts in managing transferred patients with CS initially supported by IABP. The use of tMCS for treatment of progressive CS is a growing trend over the last 2 decades, though short-term mortality rates remain persistently high.^7^, ^8^, ^9^, ^10^ To that end, accurately characterizing patients being transferred from spoke centers prior to tMCS placement is essential for gauging shock trajectory, facilitating rapid device escalation when appropriate, and timely management of complications. Notably, the authors report 10% lower in-hospital mortality rates in transferred patients who had pulmonary artery catheter data available compared with those who did not (25.6% vs 35.7%). This observation underscores the importance of early hemodynamic assessment and echoes earlier observations made by the CSWG, linking complete hemodynamic profiling in patients receiving tMCS with improved survival to discharge.^11^ Although the transfer cohort presented with fewer comorbidities, their condition deteriorated quickly, as evidenced by their higher SCAI shock stages upon presentation to the CSWG center. The rapidly progressive nature of CS has been amply demonstrated by a prior CSWG analysis, where at 24 hours, most CS patients had moved to a different CSWG-SCAI stage. In particular, stage B and C patients often progressed to a more advanced stage of shock, giving credence to the “golden day of shock” concept.^12^ Thus, one may infer that patients with CS sufficient to prompt IABP placement also deserve strong consideration for invasive hemodynamic monitoring, which may, in turn, trigger earlier referral to a tertiary center. This observation alone has far-reaching implications for patients who are deteriorating but are yet recoverable, and may therefore benefit from advanced therapies that are less likely to be available at community centers.

Given the nature of retrospective registries, however, complete data were not available for all patients. In particular, a key cohort—patients who were initially implanted with an IABP at spoke centers and deemed stable enough not to require transfer to another center—were entirely absent from the data set. This omission introduces a strong element of selection bias, as only patients who were sick enough to be transferred were included in the analysis. Indeed, the authors report that 57% of patients who were transferred presented with late CSWG-SCAI stages D to E shock, compared with 39% of patients with IABP implantation at a tertiary site. Thus, comparing the sickest of spoke patients to all-comer hub patients might, at least partially, explain the discrepancies between the groups in terms of outcomes and complication rates. Also notable is the higher proportion of HF-CS patients presenting to tertiary/CSWG sites, as prior data have suggested that HF-CS patients (vs AMI-CS patients) have lower in-hospital and 1-year mortality despite lower use of tMCS.^13^ Additionally, data on how long patients were maintained on IABP therapy at the spoke centers prior to transfer is not available, nor is the time between initial presentation at the transferring center and IABP implant. Therefore, it is unknown if these variables were partially, or perhaps even primarily, responsible for the observed differences in outcomes. Future registries should actively seek to collect these and other missing data points on transferred patients to help build better regional shock systems of care.

In conclusion, this retrospective analysis examined patients who presented with CS necessitating IABP placement at referring vs tertiary centers and found that transferred patients, despite presenting with fewer comorbidities, had higher rates of overall in-hospital mortality and major complications. Missing data notwithstanding, the findings of this analysis should strongly encourage both involvement of the referring physician on shock calls initiated for patient transfer, immediate (and ideally, complete) hemodynamic profiling at the time of tMCS placement, and expeditious escalation planning upon arrival at tertiary centers, as these variables may ultimately hold the keys to patient survival. Until we have a more detailed understanding of initial shock presentation at referring hospitals, rationale for choice and timing of tMCS implant, as well as triggers for transfer initiation, we cannot fully define the optimal care pathway for patients being transferred with CS.

## Peer review statement

Associate Editor Sandeep Nathan had no involvement in the peer review of this article and has no access to information regarding its peer review. Full responsibility for the editorial process for this article was delegated to Editor-in-Chief Alexandra J. Lansky.

## Declaration of competing interest

Michele L. Esposito has received institutional research support from Abiomed. Sandeep Nathan has received consulting fees from Medtronic, Terumo Interventional Systems, ZOLL/TherOx, Johnson & Johnson MedTech, and Magenta Medical.

## References

[1] Hernandez-Montfort J., Kanwar M., Sinha S.S. (2023). Clinical presentation and in-hospital trajectory of heart failure and cardiogenic shock. JACC Heart Fail.

[2] Berg D.D., Barnett C.F., Kenigsberg B.B. (2019). Clinical practice patterns in temporary mechanical circulatory support for shock in the Critical Care Cardiology Trials Network (CCCTN) registry. Circ Heart Fail.

[3] Wayangankar S.A., Bangalore S., McCoy L.A. (2016). Temporal trends and outcomes of patients undergoing percutaneous coronary interventions for cardiogenic shock in the setting of acute myocardial infarction: a report from the CathPCI Registry. JACC Cardiovasc Interv.

[4] Garan A.R., Kataria R., Li B. (2024). Outcomes of patients transferred to tertiary care centers for treatment of cardiogenic shock: a Cardiogenic Shock Working Group analysis. J Card Fail.

[5] Tehrani B.N., Sherwood M.W., Rosner C. (2022). A standardized and regionalized network of care for cardiogenic shock. JACC Heart Fail.

[6] Sundermeyer J., Kataria R., Garan A.R. (2026). Clinical characteristics and outcomes in intra-aortic balloon pump–supported cardiogenic shock among patients transferred to tertiary care centers. J Soc Cardiovasc Angiogr Interv.

[7] Osman M., Syed M., Patibandla S. (2021). Fifteen-year trends in incidence of cardiogenic shock hospitalization and in-hospital mortality in the United States. J Am Heart Assoc.

[8] Naidu S.S., Baran D.A., Jentzer J.C. (2022). SCAI SHOCK stage classification expert consensus update: a review and incorporation of validation studies. J Soc Cardiovasc Angiogr Interv.

[9] van Diepen S., Katz J.N., Albert N.M. (2017). Contemporary management of cardiogenic shock: a scientific statement from the American Heart Association. Circulation.

[10] Jentzer J.C., van Diepen S., Barsness G.W. (2019). Cardiogenic shock classification to predict mortality in the cardiac intensive care unit. J Am Coll Cardiol.

[11] Garan A.R., Kanwar M., Thayer K.L. (2020). Complete hemodynamic profiling with pulmonary artery catheters in cardiogenic shock is associated with lower in-hospital mortality. JACC Heart Fail.

[12] Ton V.K., Li S., John K. (2024). Serial shock severity assessment within 72 hours after diagnosis: a Cardiogenic Shock Working Group report. J Am Coll Cardiol.

[13] Sinha S.S., Rosner C.M., Tehrani B.N. (2022). Cardiogenic shock from heart failure versus acute myocardial infarction: clinical characteristics, hospital course, and 1-year outcomes. Circ Heart Fail.

